# The Updated Role of Transcranial Ultrasound Neuromodulation in Ischemic Stroke: From Clinical and Basic Research

**DOI:** 10.3389/fncel.2022.839023

**Published:** 2022-02-11

**Authors:** Shuiping Zhu, Bin Meng, Jianping Jiang, Xiaotao Wang, Na Luo, Ning Liu, Huaping Shen, Lu Wang, Qian Li

**Affiliations:** ^1^Department of Geriatric Medicine, Rongjun Hospital, Jiaxing, China; ^2^Department of Ultrasound, Rongjun Hospital, Jiaxing, China; ^3^Starbody Plastic Surgery Clinic, Hangzhou, China

**Keywords:** transcranial ultrasound stimulation, ischemic stroke, mechanism, review, limitation

## Abstract

Ischemic stroke is a common cause of death and disability worldwide, which leads to serious neurological and physical dysfunction and results in heavy economic and social burdens. For now, timely and effective dissolution of thrombus, and ultimately improvement in the recovery of neurological functions, is the treatment strategy focus. Recently, many studies have reported that transcranial ultrasound stimulation (TUS), as a non-invasive method, can dissolve thrombus, improve cerebral blood circulation, and exert a neuroprotective effect post-stroke. TUS can promote functional recovery and improve rehabilitation efficacy among patients with ischemic stroke. This mini-review summarizes the potential mechanism and limitation of TUS in stroke aims to provide a new strategy for the future treatment of patients with ischemic stroke.

## Introduction

Ischemic stroke is the most common type of cerebrovascular disease. According to the data of the global burden of disease (GBD) study, stroke is the most common cause of death among Chinese residents (GBD 2019 Stroke Collaborators, 2021). From 2010 to 2019, the incidence of ischemic stroke has increased from 129/1,00,000 in 2010 to 145/1,00,000 in 2019, and the prevalence of ischemic stroke has increased from 1,100/1,00,000 in 2010 to 1,256/1,00,000 in 2019. According to China’s aging population trend and the seventh census data, in 2021, there will be approximately 17.8 million patients with stroke, with 3.4 million new stroke patients and 2.3 million stroke-related deaths among the population over 40 years of age in China each year. China is the largest developing country, accounting for one-fifth of the world’s total population, with the highest number of patients with stroke worldwide (Wang et al., 2017). Current treatments for ischemic stroke include thrombolysis, mechanical thrombectomy, and neuroprotective therapies (Liaw and Liebeskind, 2020). However, intravenous thrombolytic therapy has a strict treatment time window, with the risk of rebleeding, making its clinical application limited.

Statistical data have shown that only 16% of patients with acute ischemic stroke in China are admitted to the hospital within 3 h of symptom onset, and only 1.3% of these patients receive thrombolytic therapy (Wu et al., 2019; Shen et al., 2020; Tu et al., 2021a,b). Transcranial ultrasound stimulation (TUS) is a non-invasive technique for patients with stroke, which can stimulate specific brain areas, and improve neural activity and connectivity. The advantages of transcranial ultrasound for brain stimulation are that it does not necessitate surgery or genetic alteration but confers spatial resolutions superior to other non-invasive methods such as transcranial magnetic stimulation (TMS; Tufail et al., 2011; Kim et al., 2012). TMS uses magnetic fields to pass through the skull to stimulate the brain tissue and has been widely used for disorders caused by the brain lesions, such as those caused in the type of depression that does not respond to medication, and cognitive impairment after stroke. The disadvantage of TMS is the lack of good spatial resolution, which results in limitations in the application of neural rehabilitation (Dionísio et al., 2018; Krogh et al., 2021; Liu et al., 2021a). The characteristics of high penetration and high spatial resolution of TUS have shown therapeutic potential in stroke treatment (Guo et al., 2015; Li et al., 2017; Liu et al., 2019; Wu et al., 2020; Malinova et al., 2021). Transcranial ultrasound is roughly divided into two types based on frequency: one is diagnostic transcranial ultrasound, with a frequency of 1.0–15 MHz, whereas the other is transcranial aggregation ultrasound, with a frequency lower than 1.0 MHz (Yang et al., 2008; Deng et al., 2021). At present, most studies focus on the thrombolytic effect and the mechanism of neuroregulation in low-frequency TUS (Li et al., 2017; Fomenko et al., 2018; Liu et al., 2019). TUS is able to transmit a certain frequency of an ultrasonic wave on the human skull with a specific ultrasonic probe. Through the skull, the ultrasonic energy is transmitted to the brain tissues, stimulating the brain to produce a series of biological effects. TUS has been explored in arterial thrombolytic therapy and post-stroke rehabilitation therapy as an emerging and non-invasive brain stimulation method (Rubiera et al., 2008; Tsivgoulis et al., 2008; Barlinn et al., 2013).

## Neuroprotective Effect of Transcranial Ultrasound Stimulation in Stroke

The neuroprotective effect of TUS has become a hot topic in recent years, especially in the field of stroke. TUS mainly exerts a neuroprotective effect through the following mechanisms.

### Rapid Restoration of Cerebral Blood Supply and Improvement of Cerebral Blood Flow

The earlier the restoration of cerebral blood supplies after acute cerebral infarction, the better the recovery of neurological function (Fisher and Bastan, 2008; Molina and Alvarez-Sabín, 2009). Therefore, improving cerebral circulation before irreversible changes occur in the brain tissue and alleviating neuronal damage are the key factors for the treatment of ischemic stroke and also have a positive significance in improving the rehabilitation efficacy post-stroke. After ischemic stroke, the neurons in the stroke center rapidly undergo apoptosis and necrosis because of ischemia and hypoxia, whereas the surrounding cells still have transient survival ability because of the existence of collateral circulation, thus forming an ischemic penumbra. Within a certain period of time, cells in this area can either undergo apoptosis or return to the normal brain tissue (Bonnin et al., 2021; Davis and Donnan, 2021; Yang and Liu, 2021). If the ischemic penumbra is rescued in time, it can effectively prevent stroke progression; further, the successful rescue of ischemic penumbra is also conducive to the recovery of the nervous system and physical function in the future. TUS has been proved to be beneficial for the improvement of cerebral blood circulation after acute ischemic stroke, and within a certain range, cerebral blood flow also shows a gradually increasing trend with the increase of stimulation intensity and duration (Yuan et al., 2020, 2021; Liu et al., 2021b). However, with an improvement in cerebral blood flow, the risk of rebleeding also increases. A previous clinical trial (Daffertshofer et al., 2005) has confirmed that low-frequency (300 kHz) TUS not only resulted in improvement of the thrombolytic efficiency of tissue plasminogen activator (tPA) but also caused an increased rate of a cerebral hemorrhage in patients concomitantly treated with intravenous tPA. The limitation of the study was that only 26 patients were included. Combined lysis of thrombus with ultrasound and systemic tPA for emergent revascularization in acute ischemic stroke (CLOTBUST-ER), an international four-center phase II trial, demonstrated that in patients with acute ischemic stroke, transcranial ultrasound augments tPA-induced arterial recanalization with a non-significant trend toward an increased rate of clinical recovery from stroke, compared with the control group. The rates of symptomatic intracerebral hemorrhage were similar between the active and control groups (Schellinger et al., 2015; Katsanos et al., 2020). Further studies are still awaited on this important issue. A previous study has shown that the earlier the transcranial ultrasound intervention, the better the neuroprotective effect (Liu et al., 2019). Therefore, early use of TUS after stroke may effectively improve the brain–blood supply, restore local blood circulation, rescue the ischemic penumbra, and ultimately reduce brain tissue damage.

Transcranial ultrasound stimulation has also been shown to improve the vascular recanalization rate, which is an important index to evaluate the treatment effect in acute ischemic stroke. Evgenii et al. (Kim et al., 2021) reported a wireless, wearable system to achieve ultrasound brain stimulation in freely behaving animals. The brain activity induced by the system was monitored as cerebral hemodynamic changes *via* near-infrared spectroscopy. The system was also applied to stroke rehabilitation after temporal middle cerebral artery occlusion (MCAO) in rats. The stimulation was found to induce hemodynamic changes in the sonicated area, whereas open-field tests showed that ultrasound applied to the ipsilateral hemisphere for 5 consecutive days after stroke facilitated recovery. Another study conducted by Wu et al. (2020) aimed to determine the neuroprotective effect of low-intensity TUS at different time points using endothelin-1-induced MCAO in rats. The results showed that the rats that received low-intensity TUS exhibited reduced damage of the affected brain tissue after cerebral ischemia. The greatest protective effect was found with ultrasound stimulation of 30 min after cerebral ischemia. Hameroff et al. (2013) found that using 8 MHz ultrasound to stimulate the upper frontotemporal cortex of patients for 15 s could increase arterial oxygen saturation, suggesting that ultrasound stimulation at a higher frequency may alleviate ischemic hypoxic changes after stroke. Furthermore, one study (Alexandrov et al., 2019) found that within 3 h after ischemic stroke onset, low-frequency transcranial ultrasound therapy within 30 min after thrombolysis can significantly enhance atenolol enzyme-induced arterial recanalization ability compared with the control group, but the results also indicated that transcranial ultrasound therapy did not significantly improve patient outcomes in 90 days after stroke.

### Reduced Inflammatory Response and Apoptosis

Inflammatory mediators can cause further damage to neurons in the ischemic penumbra. A recent study applied low-intensity TUS to the ischemic cortex after distal MCAO and found that ultrasound may activate coagulation factors through certain signal transduction pathways and reduce neutrophils in the ischemic region, thus reducing the inflammatory response and facilitating neuronal recovery in the ischemic penumbra (Guo et al., 2015). Similarly, another research on Parkinson’s disease rat model found that the low-intensity pulsed ultrasound treatment significantly inhibited 6-OHDA-induced glial activation and the phosphorylation of nuclear factor-κB p65 in the substantia nigra pars compacta. Further evaluation revealed that low-intensity pulsed ultrasound effectively preserved the levels of neurotrophic factors, dopamine transporter, and tight junction proteins in the blood–brain barrier (Song et al., 2021). Furthermore, (Zhou et al., 2021) revealed that TUS reduced the chronic inflammatory response in microglia and astrocyte activation, whereas Pang et al. (2021) found that TUS could attenuate the level of TNF-α.

As ischemic stroke induces cellular apoptosis, especially neuronal apoptosis, which leads to neurological dysfunction, determining a means to alleviate neuronal apoptosis has become an important issue for researchers while evaluating the outcome of patients with ischemic stroke. A study conducted in 2021 (Zhou et al., 2021) found that TUS could reduce the level of apoptosis-related protein Bax, and improved the movement and learning in aging rats. Su et al. (2017) further observed that low-intensity pulsed ultrasound could inhibit the progression of apoptosis following traumatic brain injury. Thus, the neuroprotective effects of TUS may be associated with the TrkB/Akt-CREB signaling pathway. Another study from the same team (Chen S. F. et al., 2018) found that the low-intensity pulsed ultrasound significantly attenuated the brain edema and neuronal death, reduced neutrophil infiltration and microglial activation, increased the Bcl-2/Bax ratio, and enhanced the phosphorylation of Bad and FOXO-1, ultimately improving the functional outcomes. These results indicated that the neuroprotective effects of TUS are associated with a reduction of early inflammatory events and inhibition of apoptotic progression.

### Promotion of the Release of Neurotrophic Factors

Neurotrophic factors, such as brain-derived neurotrophic factor (BDNF) and glial cell line-derived neurotrophic factor (GDNF), are considered to be involved in the regulation of key nerve functions and neuroplasticity in stroke (Wang et al., 2006; Luo et al., 2019; Liu et al., 2020). A previous study has found that TUS effectively prevents cerebral ischemia/reperfusion injury through apoptosis reduction and BDNF induction (Chen C. M. et al., 2018). Su et al. (2017) observed that low-intensity pulsed ultrasound could increase the BDNF protein levels following a traumatic brain injury. The neuroprotective effects of TUS may be associated with the enhancement of protein levels of neurotrophic factors. Similarly, another study conducting MCAO using a C57BL/6J mouse model found that the low-intensity pulsed ultrasound accelerated the expression of BDNF in the brain of stroke mice and significantly moderated neuronal function after injury including neurological score, motor activity, and brain pathological score. In addition, Yang et al. (2015) demonstrate that TUS could enhance the protein levels of neurotrophic factors (i.e., BDNF, GDNF, VEGF, and GLUT1), which could have neuroprotective effects against neurodegenerative diseases. In a study using an ischemic stroke mouse model, low-intensity pulsed TUS could induce BDNF expression, decreasing the percentage of damaged neurons and the loss of neurological function after stroke. Tsai et al. (Zhang et al., 2019) interestingly found that TUS can also exert antidepressant-like effects. TUS could change the expression of BDNF in the hippocampus of rats. Given that BDNF plays an important role in the pathogenesis of depression, promoting BDNF could have a therapeutic effect (Hashimoto, 2010).

### Thrombolytic Effect of Transcranial Ultrasound Stimulation

Ultrasound has direct and indirect thrombolytic effects. Indirect thrombolytic effects of TUS mainly enhance the effectiveness of thrombolytic drugs. In addition, ultrasound can directly dissolve the thrombus through its special physical and chemical properties (Cao et al., 2021; Doelare et al., 2021; Mei and Zhang, 2021). The main thrombolytic effects of TUS are summarized as follows.

#### Cavitation Effect

Cavitation is the generation of a large number of small bubbles when ultrasound is applied to a liquid (Lahiri et al., 2021). These bubbles rapidly vibrate, expand, and burst. A large amount of energy is released at the moment of bubble explosion, causing the thrombus to tear and decompose, exposing the surface of the thrombus in large quantities, and further accelerating the dissolution of the thrombus (Ma et al., 2020; Jo et al., 2021; Singh et al., 2021). The cavitation-induced thermal effect has been confirmed as a potential mechanism underlying this phenomenon. When a certain amount of heat is generated at the moment of bubble expansion, the heat effect may be the main role of ultrasound therapy, because a certain amount of heat effect can increase the activity of fibrinolytic enzymes, which is conducive to its binding with thrombosis (Wu et al., 2020). Intensity-focused ultrasound can generate high heat for the ablation of tumors and other biological tissues (ter Haar, 2007; Bessonova et al., 2010; Simon et al., 2012). The mechanism underlying this is complex. Some studies have proposed that high-intensity focused ultrasound can affect the action potential of axons (Wahab et al., 2012); the electrical activity of neurons can be suppressed by disrupting the ultrastructure of synapses by blocking the connections between them (Borrelli et al., 1981; Tsui et al., 2005). Furthermore, the thermal effect of high-frequency and high-intensity focused ultrasound can damage nerve tissue and thus block transmission between synapses (Foley et al., 2008; Colucci et al., 2009). In contrast, one research found that although low-intensity TUS was eventually converted into heat energy when it passed through non-ideal media, the ultrasonic signal had almost no thermal effect on the brain tissue in the process of ultrasonic stimulation. The heat is extremely weak, much lower than the predicted heat required to produce obvious biological effects of temperature threshold (Liu et al., 2019). The differences in the abovementioned studies may be related to the differences between animal models and clinical patients. However, it is still controversial whether low-frequency TUS has a meaningful thermal effect during treatment. Whether the strength of the thermal effect is related to the frequency, intensity, and duration of TUS, and whether the thermal effect will cause damage to the brain tissue, needs further research.

#### Biomechanical Effect

The mechanical action is the main mechanism of ultrasound thrombolysis. The mechanical vibration of ultrasound can destroy blood clots, decompose thrombi, increase the contact between enzyme and fibrin, and promote the dissolution of thrombi. Ultrasound can also enhance the vitality of the brain cells and promote the repair of nerve cells after cerebral ischemia (Masomi-Bornwasser et al., 2021; Shin Low et al., 2021).

#### Microflow Effect

The microflow effect is caused by the cavitation effect. Here, the pressure generated by the burst microbubble causes the liquid to form microflow, which can accelerate drugs to the ischemic region (Matsievskii, 2003; Park et al., 2019; Li et al., 2021).

## Limitations of the Current Transcranial Ultrasound Stimulation Study

Treatment with TUS lacks clinical trials, as there are great differences between animal models and clinical patients, many problems remain to be solved in the clinical application of TUS. Although some clinical studies have proved that TUS can improve the clinical efficacy in ischemic strokes, these evaluation methods cannot reflect the improvement in a patient’s ability during the rehabilitation process. More detailed and specific assessment scales should be used for evaluating the effect of TUS, such as assessment of cognitive, speech, sensory, motor, and other aspects. Moreover, most of the current clinical studies are about a combination of TUS and thrombolytic drugs to improve the thrombolytic effect. Clinical research in the future may explore TUS and other rehabilitation treatment methods, such as physical therapy, speech therapy, and low-frequency neuromuscular electrical stimulation, to improve the effectiveness of rehabilitation therapy in patients with ischemic stroke. For example, anodal transcranial direct current stimulation combined with constraint-induced movement therapy resulted in the improvement of functional ability of the paretic upper limb compared with constraint-induced movement therapy alone, indicating that TUS may enhance the effect of rehabilitation in patients with chronic stroke (Figlewski et al., 2017). In addition, there is no clear standard answer with regard to the specific parameters of TUS. Previous studies found that transcranial ultrasound with different frequencies can produce different excitatory or inhibitory effects on the brain (Krishna et al., 2018; Zhang et al., 2021). The ultrasonic stimulation parameters mainly include frequency, pulse repetition frequency, duty cycle, pulse duration, and ultrasonic intensity (Uddin et al., 2021). Frequency refers to the number of oscillation cycles per unit time, importantly, as the frequency is inversely proportional to the wavelength, the higher the frequency, the smaller the focal spot volume, and the more significant the acoustic attenuation and scattering effect. Therefore, it is necessary to explore the most suitable frequency and intensity of TUS according to the location depth of the stimulation target and the thickness of penetration through the skull, and the safety of different parameter combinations in clinical application.

## Conclusion

**FIGURE 1:**
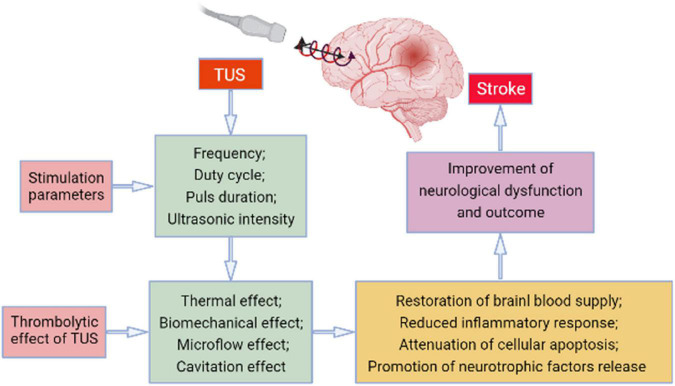
The potential mechanism involved in the transcranial ultrasound stimulation (TUS)-induced effect on stroke.

Ultrasound thrombolysis has already been used widely; low-frequency TUS can exert its effect based on the mechanical vibration, cavitation, or microflow, consequently dissolving the thrombus, reducing the infarct size in patients, promoting cerebral circulation, rescuing the ischemic penumbra, and improving the prognosis of patients with stroke. Low-frequency TUS is mostly used clinically at present. Numerous studies have explored the mechanism of low-frequency TUS and confirmed that low-frequency TUS has certain neuroregulatory effects, which have both excitatory and inhibitory effects on the human cerebral cortex (Figure 1).

In summary, although there are still many issues that need to be explored and solved in terms of clinical application, numerous studies have shown that TUS can promote thrombolysis, increase cerebral blood circulation, and improve neurological recovery. Thus, after ischemic stroke onset, timely and proper TUS intervention may improve the neurological function and quality of life of post-stroke patients.

## Author Contributions

QL participated in the design of the review. SZ, BM, JJ, XW, NLu, and NLi drafted the manuscript. HS and LW critically revised the text and figure. All authors read and approved the final manuscript.

## Conflict of Interest

The authors declare that the research was conducted in the absence of any commercial or financial relationships that could be construed as a potential conflict of interest.

## Publisher’s Note

All claims expressed in this article are solely those of the authors and do not necessarily represent those of their affiliated organizations, or those of the publisher, the editors and the reviewers. Any product that may be evaluated in this article, or claim that may be made by its manufacturer, is not guaranteed or endorsed by the publisher.
